# Lyme Disease Forecasting in the U.S. Department of Defense: Summary of One- to Three-Month Forecasts, January–October 2024

**Published:** 2026-01-26

**Authors:** Mark L. Bova, Sneha P. Cherukuri, Shaylee P. Mehta, Christian T. Bautista

**Affiliations:** Integrated Biosurveillance Branch, Armed Forces Health Surveillance Division, Public Health Directorate, Defense Health Agency, Silver Spring, MD

Lyme disease incidence has been increasing among U.S. Department of Defense (DOD) service members over the past 20 years, threatening the health and readiness of the force. ^1^ Syndromic surveillance of tick bite visits can provide timely information that might predict changes in tick-borne disease incidence and geographic spread. ^2^ Tick and tick-borne illness surveillance programs in the U.S. face many barriers, however, including inconsistent funding and limited capacities. ^3^

Epidemic forecasting models can be used to enhance routine surveillance and inform public health policy. ^4^ Previous research on Lyme disease forecasting has focused on general time series and machine-learning models; however, these models have demonstrated high percentage errors and limited predictive accuracy. ^5 , 6^ Further research is needed to explore alternative modeling approaches that may improve forecasting performance.

Since 2019, the Armed Forces Health Surveillance Division, Integrated Biosurveillance (AFHSD-IB) Branch, within the Defense Health Agency's Public Health Directorate, has been conducting respiratory disease forecasting using syndromic surveillance data. ^7^ Since 2024, AFHSD-IB has integrated vector-borne disease forecasting to provide situational awareness and inform public health responses from DOD senior leaders. This report aims to predict the number of future Lyme disease and tick bite encounters among U.S Military Health System (MHS) beneficiaries using outpatient encounter data and multiple forecasting methods.

## Methods

Tick bite encounters were used as a proxy for tick exposure, while Lyme disease encounters served as a proxy for Lyme disease diagnoses in the absence of trusted case reporting. Encounter definitions were developed using internal criteria.

A single instance of a Lyme disease encounter was defined using the International Classification of Diseases, 10th Revision (ICD-10), discharge diagnosis code ‘A69.2’ and chief complaint terms “Lyme disease,” “erythema migrans,” or “bulls-eye rash,” or their misspellings, in the chief complaint field; any mention of a history of Lyme disease was excluded. Similarly, tick bite encounters were defined as health records mentioning “tick bite” and associated misspellings (including “tic” or “tik” for tick and “bit” for bite) in the chief complaint field. Monthly direct-sourced outpatient encounter data for each military treatment facility were obtained from the DOD's Electronic Surveillance System for the Early Notification of Community-based Epidemics (ESSENCE). Data were then aggregated into 4 U.S. surveillance regions, selected based on their high volume of Lyme disease encounters in 2024: National Capital Region, New England, Tidewater, and West Point. Surveillance regions are collections of neighboring military installations, clinics, and hospitals used for disease surveillance and reporting. (Information about each surveillance region is shown in **Supplementary Table 1** .)

Monthly encounter data from January 2021 through October 2024 were collected and used to generate 1-through 3-month forecasts for each outcome metric and surveillance region for the period January through October 2024. Models were trained each month, using data from January 2021 through the most recent month.

Several time series and machine-learning models were used for forecasting. The seasonal autoregressive integrated moving average (SARIMA) model is based on the widely used Box-Jenkins method for univariate time series forecasting. ^8^ The error, trend, seasonal (ETS) model is another time series model that belongs to a special class of exponential smoothing models known as state-space models. ^8^ The exponentially weighted moving average (EWMA) model is a highly sensitive model that effectively identifies subtle data pattern changes due to its weighting scheme, which is of particular importance when assessing rare outcomes. ^9^ The vector autoregressive (VAR) model is a forecasting algorithm that can be used when 2 or more time series influence each other. ^10^ The neural network (NNET) model is a machine-learning model that can adapt to changing inputs, generating the best possible results without needing to redesign the output criteria. ^11^ Prophet is open-source forecasting model created by Facebook that allows users to easily customize and produce high quality forecasts. ^12^ Finally, a baseline naïve model was created using data from the previous tickborne season. An ensemble (ENS) model was also calculated as the average of all individual model forecasts.

A log-transformed weighted interval score (WIS) was used to measure accuracy of the model forecasts. WIS was previously established as a scoring method for respiratory disease forecasts, ^13^ with lower values indicating more accurate forecasts. The median absolute percentage error (MAPE), another common metric for forecasting error, was also calculated. All analyses were performed in R software 4.4 (R Foundation for Statistical Computing, Vienna, Austria), including the *fable* ^14^ and *fabletools* ^15^ packages.

## Results

**FIGURE 1.**
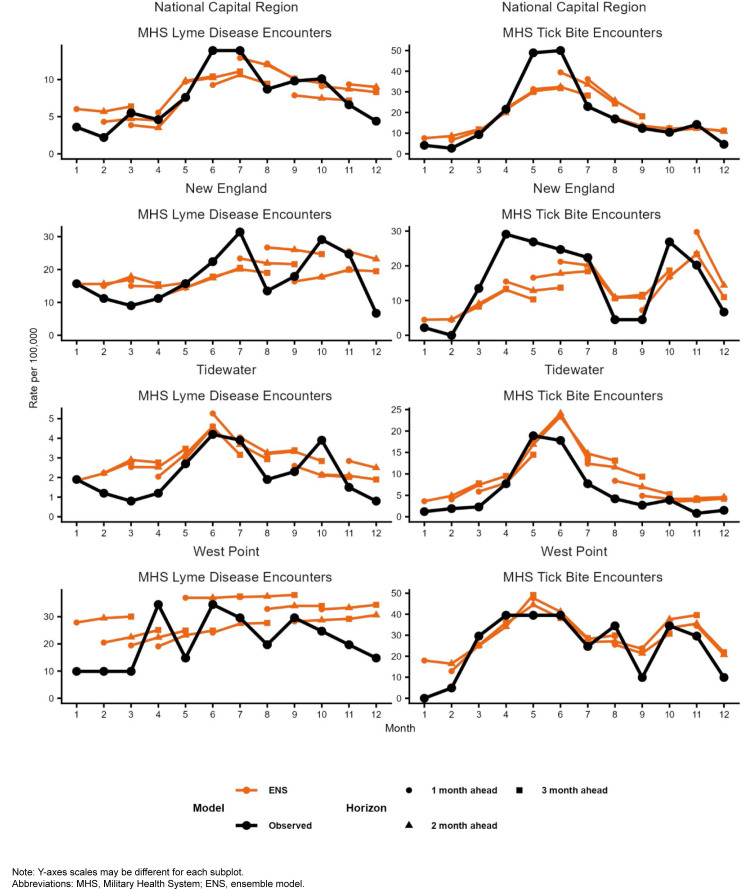
Ensemble Model Observed and Predicted Values, by Surveillance Region and Outcome Metric, 2024

Our analyses indicated that observed Lyme disease encounters increased from late winter to early spring, peaking during the summer months, except for West Point, which peaked in April. West Point also had the highest peak (34.5 per 100,000 MHS beneficiaries), followed by New England (31.4 in July), National Capital Region (13.9, June), and Tidewater (4.2, June) **(Figure 1)** . Observed tick bite encounters increased from February until spring for each surveillance region. The National Capital Region had the highest peak (50.0 per 100,000 MHS beneficiaries in June), followed by West Point (39.5, April), New England (29.1, April), and Tidewater (18.9, May). **Figure 1** also shows 1- through 3-month ahead horizon forecasts for the ENS model.

**FIGURE 2.**
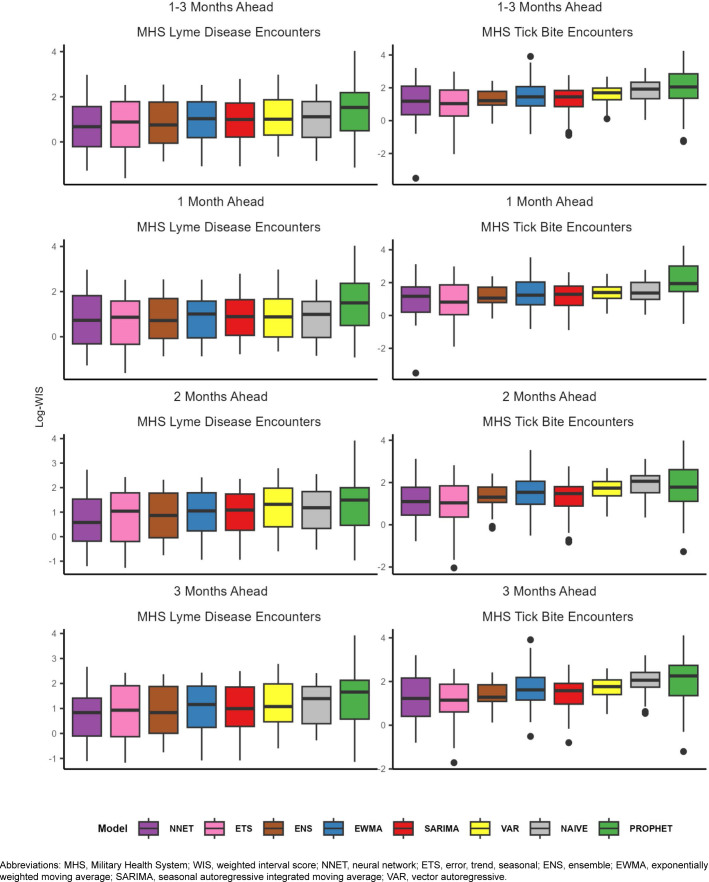
Median Log-Weighted Interval Score by Forecasting Horizon, Outcome Metric and Model, 2024

For Lyme disease encounter forecasts, the NNET model had the lowest median log-WIS across the 2-month (0.6) and 3-month (0.8) ahead horizons, while the ENS model had the lowest median log-WIS for the 1-month ahead horizon (0.7) **(Figure 2)** . The ETS model had the lowest MAPE for Lyme disease encounters for the 1-month ahead horizon (33%), while the NNET model had the lowest MAPE for the 2-month ahead horizon (37%), and the ENS model had the lowest MAPE for the 3-month ahead horizon (28%) ahead horizon **(Supplementary** **Figure 2)** . During the months with the highest activity (March–October), MAPE for the ENS model improved to 29% and 27%, respectively, for the 1-month and 3-month ahead horizons, while the MAPE for the NNET model improved to 29% for the 2-month ahead horizon, indicating greater forecasting accuracy during high activity periods when compared to the total surveillance time ( **Supplementary Table 2** ).

For tick bite encounter forecasts, the ETS model had the lowest score (1.0) for all horizons, as well as for the 1-(0.8), 2-(1.0), and 3-month (1.2) ahead horizons, followed by the NNET model (1.2, all horizons; 1.2, 1-month; 1.1, 2-month; and 1.2, 3-month) **(Figure 2)** . The ETS model had the lowest MAPE across all 1-month (25%), 2-month (23%), and 3-month (24%) ahead horizons **(Supplementary** **Figure 2)** . During the months of highest activity, MAPE for the ETS model improved to 21%, 22%, and 24% respectively for the 1-month, 2-month, and 3-month ahead horizons ( **Supplementary Table 2** ).

## Discussion

This study produced 2 major findings. First, among all the time series and machine-learning models mentioned in our analysis, 3 models—ENS, ETS, NNET—provided the most accurate forecasts of Lyme disease and tick bite activity in MHS beneficiaries, with increased accuracy during peak activity periods. This finding suggests that these 3 models would be valuable as early warning signals for public health surveillance and preparedness. It is important to note, however, that model accuracy decreased at longer horizons. Second, MAPE estimates were higher than those reported in previous studies using SARIMA models, although accuracy was improved by focusing on periods of higher activity. Individual surveillance regions aligned with the findings of the aforementioned study. ^6^

Limitations of this study must be considered. First, data lags and inconsistent reporting of Lyme disease cases within the MHS prevented validation of encounter data against confirmed case data. Consequently, sensitivity analysis of encounter definition robustness could not be performed, and therefore, results may not be representative of overall disease incidence. The training data set was derived from a 3-year period of encounter data, but additional historical data may be needed to properly train some of the models. Due to limited technology capacity, particularly hardware, this study also did not utilize more computationally intensive machine-learning models, such as long short-term memory and random forest models, potentially limiting the accuracy of the forecasts. In addition, these models are susceptible to over-fitting when there is insufficient training data.

Despite these limitations, this study provided the first quantitative evidence of the use of outpatient encounter syndromic surveillance data for forecasting Lyme disease and tick bite encounters in the MHS population. Lyme disease forecasting can provide vital information for anticipating the impact on military health and readiness, as well as informing effective public health responses and mitigation efforts within the DOD. Further research is required to explore additional models, more robust training data, and other covariates, including incident Lyme disease cases and other key predictors, such as host species, geographic factors, climate, and weather.
